# Observations on the Propriety of Adopting, in Great Britain, a Plan of Medical Police

**Published:** 1807-12

**Authors:** John Roberton

**Affiliations:** Surgeon, Edinburgh


### *Observations on the Propriety of adopting, in Great Britain, a Plan of Medical Police.* By John Roberton, Surgeon, Edinburgh.

MANY attempts have been made by philosophers to account, in a general manner, with respect to climate, situation, &c. for the great mortality which prevails in this and in every other country, so far as these are concerned. But the little benefit that has arisen from their researches in this very important branch of science, is a convincing proof proof that their labours have been unattended with success.

Although I have considered the failure of this important object with the deepest regret, I have not yet ranked it in the list of those irremediable evils, which hold human ingenuity at defiance; but have, for some time past, employed myself in collecting materials relative to it, which, if my success answers my expectations, I may probably be induced to lay before the public, under the title of "*A Plan of Medical Police*," in a more detailed, and consequently more interesting form, than the present Essay.

I have never been of opinion that a work ought to be completely abandoned, because it cannot at first be brought, in every respect, to a state of the greatest perfection; or rendered sufficiently accurate in selection and arrangement, to pass the fiery ordeal of those gentlemen, who style themselves *Reviewers;* and who, if they would, in general, give that encouragement to rising merit, which it really deserves, and which, from the nature of their employment, they ought to do, I am convinced the world would, at this moment, be in possession of many original and important observations, which might have done honour to literature in any age.

I am sorry to say that thus, by indiscriminately precipitating, in the same chaos, the most common uningenious productions of those I would call mere artists in literature, with the works of genius in pursuit of honest fame, must often injure, but never can benefit, their country by their exertions. On this account perhaps it is, that genuine productions are not often to be met with; so seldom, indeed, that if in every volume that has hitherto been written, a single original 6pinion could be found, our stock of information would be far greater than that which we at present possess.

I am well aware that the general arrangement of such a plan, as it may be necessary to follow upon this interesting subject, is replete with difficulty; and that the particular one which I have here indicated may, in many points, be susceptible of improvement; but, as it will ultimately be productive of numerous benefits, as far as health is concerned, it must soon become a subject of great national importance. If, indeed, even the life of a single individual is preserved by my exertions, I shall be contented, in the expectation that others, improving the principle, may at length bring my plan to a state of greater pertion.

Although,

Although, in the consideration of a plan of such importance, we are at first unable to accomplish all those views, which may be necessary to render it perfect, we ought not, on that account, to allow our exertions to become paralyzed for want of attempts; but, without resting satisfied with what has already been done, we ought, by concentrating every energy of our mind, either in more scientifically following the doctrines already sanctioned by public opinion, or in framing or adopting others, constituted upon principles more evidently calculated to alleviate human distress, to realize endeavours of such universal importance.

Many of my opinions, on this subject, must long have been familiar to every one; as several of those causes, which first induced me to think seriously about it, have been acknowledged sources of disease, from the very earliest periods of history; but so far as I am acquainted with the writings of former times, no author has been fortunate enough, either from incorrect statement of facts, or from deficient or erroneous modes of reasoning, to induce the legislative body of this country, to adopt a general systematic plan for their prevention.

The principal general sources of disease in this, and perhaps every other country, I believe, with very few exceptions, to exist in external, and for the most part removable causes; but, from our familiarity with numberless circumstances, which are unquestionably injurious to our comforts, and even destructive to our constitutions, we, in the common bustle of life, insensibly become so to overlook them, as scarcely ever to regard them in a just point of view.

The fenny counties of England, and other swampy grounds, most of which, though drainable, are suffered to exist—the local situation, and the particular construction of dwelling-houses—the sources of uncleanness, which, in every part of our island, obtrude themselves upon our notice, are all, (and perhaps the principal) sources of disease. In other countries diseases, most destructive in their nature, arise from similar causes; particularly in warm climates, where putrefaction almost immediately follows the death of either animal or vegetable matter. Even diseases must be expected to arise from sources peculiar to certain latitudes; and wherever disease is found to arise from any derangement in natural objects, or indeed from any general cause, it may, even in the present imperfect state of physics, though perhaps with difficulty, be removed.

moved. But as I propose to confine my observations to what principally relates to our own country, I shall not enter upon this part of the task: yet it is worthy of attention, and I hope other persons will be induced to consider it.

In earlier times, all these causes must have greatly contributed to the propagation and frequency of disease; and although history informs us of uncommon feats of strength performed by individuals, in former times, yet from this we can draw no conclusion, respecting the health of ancient nations in general. For I believe that, in every part of the world, the greatest proportion of its inhabitants have passed their lives in obscurity; and although employed in the most useful pursuits, their names and their diseases are equally unknown to us—they are only rare occurrences, magnified by tradition, that have been transmitted to us.

I may remark, that the great improvements which have lately taken place in the internal structure of houses, in several parts of our island, have not been the result of accurate reasoning, either on the part of the builder, or of the proprietor, but solely that of luxury.

If the poor alone were sufferers from the operation of such causes, the wonder would not be so great, that nothing has been done for their prevention; but the rich are often equal sufferers. I do not mean to say that they are so in every respect. They are not like those cold and hungry wretches, who, in general, experience a paroxysm of joy only, when in a state of intoxication; and who are obliged to hovel together in obscure cheerless garrets, hardly protected even from the severity of the winter's storm, or who are compelled to groan out their existence in lothsome cellars, and to breath an atmosphere impregnated with every quality that is destructive of human life; where, in general, moral principles are as much corrupted, as physical powers are destroyed; where the young of our own species are, at once, tainted with disease, and trained from infancy in the exercise of every thing that is vicious, who, if they had been placed in more favourable circumstances, and exposed to more favourable moral opportunities, might possibly have done honour to humanity.

Thousands of parents, in the situations here alluded to, whose principles, both moral and religious, have (if I may use the term) been starved out of them, distort the limbs, or destroy the eyes, of their infants, that they may thus be exposed in the public streets, and rendered objects for exciting exciting the pity, and means for extorting the money of passengers.

While the community, in general, can only survey the black outline of human distress, without even suffering themselves to attach to it that pity which is really its due, Medical Practitioners, particularly in large and populous cities, are daily, while prosecuting the duties of their profession, obliged to witness indescribable scenes of misery and wretchedness.

That the removal of many such nuisances, as I have mentioned above, the cause of all these calamities, should never have been thought of, particularly in Great Britain, where scientific research constitutes so prominent a feature of the national character, has to me often been a matter of surprize and wonder. The adoption of such a plan would not only contribute to the happiness of individuals, but to the increase of the public revenue; as the healthy state of a country is one of the greatest—is indeed the first—cause of its prosperity and affluence.

The wealth of all nations arises from their quantity of productive labour; and, therefore, whatever proportion of unproductive individuals exist in any country, not only is an equal proportion of wealth lost to it, but an additional proportion of its wealth is destroyed. Mr. Malthus has proved, that an increase, even of active population, would, at the present moment, be prejudicial to Britain. How infinitely more so must an increase of inactive population be, not only as affording no aid to the revenue from the taxation of labour, or of income, and as being burthensome to their respective parishes, but as more completely exceeding the means of subsistence!

In order to establish a proper plan of this kind, we must be guided by experience and induction; to which the recital of the following may, in some measure, contribute.

Sometime ago, I witnessed the situation of several poor families in one neighbourhood, rendered unhealthy by the earth at the back of the houses being piled several feet above the level of their beds. This, I may mention, as a solitary instance of *one* source of disease; and, I have no doubt, that many of my professional brethren are in possession of facts, equally strong in support of it.

I was called to visit a poor man, a weaver by trade, who, I was informed, had been for the most part confined to bed for several months, being unable to follow his occupation, cupation, from a variety of complaints. His family consisted of a wife and four helpless children, who were destitute of every assistance, except what was given them by their charitable neighbours; and by this, with the addition of a scanty allowance (about 1s. 6d. or 2s. per week) allowed them by an institution, established to assist in the relief of the indigent, they were enabled barely to exist. In this state the father lay on a miserable couch, many of his joints unable to perform their office, from repeated rheumatic attacks, and frequent febrile exacerbations, and completely deprived of that nourishment which his situation required.

Several other families, in the same neighbourhood, were, from causes somewhat similar, reduced to a state of extreme misery. In some houses, the father and mother were confined to bed by disease, while their infant families were almost perishing for want of food, or compelled to beg for their daily sustenance in the common streets. In others, one or more of the children laboured under disease. The apartments of all these houses were small; they were not particularly remarkable for their dampness, but there was a rank and disagreeable smell, perceived by a person on entering them, which was much worse than that of a room in which sick people are, in general, assembled. I ordered these unfortunate people to be immediately removed to another house, better constructed, in every respect, than the former. The younger part of the family soon recovered; those advanced in life recovered slowly, and not completely.

These are sights which must, at all times, create in the feeling mind, the most distressing sensations, and ought to call forth every energy of our nature for their relief.

Therefore, I propose that a Medical Man, Clergyman, or other respectable person, in every parish throughout the country, should be solicited to give a report of the diseases peculiar to his respective district; alluding chiefly to such as may arise from general causes, and stating those causes, which seem to them to exist for them. As from these data, I conceive it would not be difficult to organize *a Plan of Medical Police*, and lay a basis for the removal of all the general causes of disease. Such a proposition cannot too speedily be carried into effect.

